# Why Is It?

**Published:** 1880-10

**Authors:** 


### WHY IS IT?

A professional brother, who has been giving some attention to the subjects, says that while softened dentine will in favorable cases, at least, solidify beneath a good filling of exy chloride of zinc it will not do so under exy-phosphate fillings. This, if true, is an interesting and important matter. Let those who know anything on the subject speak out. Such experiments require intelligent and close observation.

